# Positionspapier: Operation und Diabetes mellitus (Update 2023)

**DOI:** 10.1007/s00508-022-02121-z

**Published:** 2023-04-20

**Authors:** Antonia-Therese Kietaibl, Joakim Huber, Martin Clodi, Heidemarie Abrahamian, Bernhard Ludvik, Peter Fasching

**Affiliations:** 1 5. Medizinische Abteilung für Endokrinologie, Rheumatologie und Akutgeriatrie, Klinik Ottakring, Wien, Österreich; 2 Interne Abteilung mit Akutgeriatrie und Palliativmedizin, Franziskus Spital, Standort Landstraße, Wien, Österreich; 3 ICMR – Institute for Cardiovascular and Metabolic Research, Johannes Kepler Universität Linz, Linz, Österreich; 4 Abteilung für Innere Medizin, Konventhospital der Barmherzigen Brüder Linz, Linz, Österreich; 5 Privates Institut für Medizin & NLP, Wien, Österreich; 6 1. Medizinische Abteilung für Diabetologie, Endokrinologie und Nephrologie, Klinik Landstraße, Wien, Österreich

**Keywords:** Diabetes mellitus, Perioperatives Management, Orale Antidiabetika, Glykämische Kontrolle, Insulintherapie, Diabetes mellitus, Perioperative management, Oral antihyperglycemic therapy, Glycemic control, Insulin therapy

## Abstract

Das vorliegende Positionspapier beschreibt die Sicht der Österreichischen Diabetes Gesellschaft hinsichtlich des perioperativen Managements von Menschen mit Diabetes mellitus auf Basis der verfügbaren wissenschaftlichen Evidenz. Dabei wird Bezug genommen auf die präoperative Begutachtung und Vorbereitung sowie auf die perioperative Stoffwechselkontrolle mittels oraler Antidiabetika und/oder injektabler Therapie (Insulin‑/GLP-1-RA-therapie).

## Abstract (en)

This position statement reflects the perspective of the Austrian Diabetes Association concerning the perioperative management of people with diabetes mellitus based on the available scientific evidence. The paper covers necessary preoperative examinations from an internal/diabetological point of view as well as the perioperative metabolic control by means of oral antihyperglycemic and/or insulin therapy.

## Einleitung und Vorbemerkung

Das Thema „Operation und Diabetes mellitus“ umfasst die perioperative Periode des Managements von Menschen mit Diabetes mellitus. Perioperativ beinhaltet die präoperative (vor der Operation), intraoperative (während der Operation) und postoperative (nach der Operation) Phase. Hierbei wird insbesondere auf die präoperative Evaluierung des Gesundheitsstatus der Menschen mit Diabetes mellitus, die präoperative Stoffwechselkontrolle und das perioperative Management inklusive medikamentöser Diabetestherapie und mögliche Komplikationen eingegangen. Nachdem es zu einem globalen Anstieg der Diabetes Prävalenz und Inzidenz kommt, ist konkordant auch die Anzahl von Patient:innen mit Diabetes, die eine Operation brauchen, zunehmend [1, 2].

Zu zahlreichen wichtigen Fragestellungen dieses Themenkomplexes liegen nur vereinzelte oder gar keine randomisierten prospektiven Studien bzw. Metaanalysen vor. Im Rahmen der prospektiven, multizentrischen MOPED Studie sollen klinisch relevante Fragestellungen aus anästhesiologischer Perspektive hinsichtlich des perioperativen Managements von Menschen mit Diabetes aufgearbeitet werden, da insbesondere uneinheitliche internationale Empfehlungen den Umgang erschweren [3]. Die folgenden Erörterungen bzw. Empfehlungen beziehen sich daher auf in der Literatur verfügbares Expert:innenwissen (u. a. Buchbeiträge, Übersichtsartikel, Leitlinienempfehlungen einschlägiger Fachgesellschaften, Originalarbeiten) und auf die klinische Erfahrung der o. g. Autor:innen. Das folgende Papier kann daher keinen „imperativen“ Leitliniencharakter haben, sondern stellt ein Positionspapier der Österreichischen Diabetes Gesellschaft dar, welches versucht, das vorhandene klinische Wissen bestmöglich zusammenzufassen. Beim perioperativen Management von Menschen mit Diabetes muss jeweils auf das Individuum und die jeweilige Operation abgestimmt werden. Aus diesem Grund beinhaltet das Positionspapier nur grobe Anhaltspunkte für das klinische Handeln, kann jedoch im Einzelfall keine „Rechtssicherheit“ vermitteln.

## Präoperative Evaluierung

Die präoperative Evaluierung eines Menschen mit Diabetes mellitus ist prinzipiell analog zu nicht diabetischen Patient:innen zu sehen. Der Stellenwert internistischer „Operationsfreigaben“ wird kontroversiell gesehen, da die primäre Verantwortung für die Durchführung eines operativen Eingriffes bei den behandelnden Chirurg:innen und narkoseführenden Anästhesist:innen liegt. Zusammen mit den Operateur:innen wird das perioperative Gesamtrisiko der Patient:innen beurteilt und gemeinsam das optimale chirurgische und anästhesiologische Vorgehen definiert [4].

Die aus Sicht der Österreichischen Diabetes Gesellschaft gebotene präoperative internistische Voruntersuchung des Menschen mit Diabetes mellitus soll in erster Linie den allgemeinen Gesundheitsstatus dokumentieren und feststellen, ob vorbestehende Gesundheitsstörungen oder Therapien eine absolute oder relative Kontraindikation für den geplanten Eingriff darstellen. Gegebenenfalls ist die internistische Ausgangssituation bzw. laufende medikamentöse Therapie bezüglich des geplanten Eingriffs zu optimieren.

Die Indikation zur Durchführung eines Akuteingriffes bei vitaler Bedrohung ergibt sich naturgemäß aus der Zusammenschau der klinischen Situation.

Menschen mit Diabetes mellitus haben ein höheres Risiko für Begleiterkrankungen als gleichaltrige Personen ohne Diabetes. Das Risiko für eine bestehende Multimorbidität steigt allgemein mit dem Lebensalter und insbesondere mit der Diabetesdauer. Zu erwartende Begleiterkrankungen betreffen insbesondere das kardiovaskuläre System (z. B. koronare Herzkrankheit, zerebrovaskuläre Durchblutungsstörung, periphere arterielle Verschlusskrankheit), die Nieren und das urogenitale System (z. B. Nierenfunktionseinschränkung in Folge von Nephropathie, Infektionen) sowie die Nerven und Sinnesorgane (periphere und autonome Neuropathie, Retinopathie und Makulopathie) [5–8]. Weiters bestehen häufig Zusatzerkrankungen im Sinne des metabolischen Syndroms (z. B. arterielle Hypertonie und Hyperlipidämie) [9, 10].

Prinzipiell ist das Ausmaß der präoperativen Evaluierung und Abklärung abhängig von der Größe und Schwere des geplanten operativen Eingriffes sowie der bestehenden bzw. klinisch geschätzten Multimorbidität [4].

In der Regel umfasst eine internistische präoperative Evaluierung eine Erhebung des klinischen Status der Patient:innen (Schwerpunkte: Herz, Lunge, Carotiden, Extremitäten inklusive Blutdruckmessung an beiden Armen und Pulsstatus der Beine). Neben einer erweiterten Diabetes-spezifischen Anamnese (Diabetestyp, Therapieregime, Frequenz von Hypo- und Hyperglykämien, Hypoglykämiewahrnehmungsstörung, diabetesassoziierte Spätkomplikationen), bietet die Bestimmung von Routineparametern (komplettes Blutbild, Entzündungsparameter, Nierenfunktionsparameter inklusive Elektrolyte, Leber- und Lipidbefunde, basales TSH, Harnbefund inklusive Albumin-Kreatinin-Ratio, Gerinnung) eine gute Abschätzung des Gesundheitszustandes. Zudem ist die Bestimmung des Hämoglobin A1c (HbA1c)-Wertes (sofern nicht innerhalb der vergangenen drei Monate erfolgt) und der Blutglukosekonzentration (nüchtern oder postprandial bzw. selbsterhobenes Profil oder alternativ das ambulante Glukose Profil (AGP) von kontinuierlichen Glukosemesssystemen (CGM)) unabdingbar [4, 7, 11]. Anamnestisch ist das Auftreten von Hypoglykämien abzufragen und hier insbesondere hinsichtlich einer möglichen Hypoglykämiewahrnehmungsstörung zu explorieren [12, 13]. Die Ableitung eines Zwölfkanal-EKGs in Ruhe ist empfehlenswert bzw. bei entsprechender Anamnese und Klinik erforderlich [4, 14].

Weiterführende präoperative Untersuchungen (Thoraxröntgen, Echokardiographie, Sonographie der Carotiden, Ultraschalluntersuchung des Abdomens inklusive Nieren, Ergometrie, bildgebende Diagnostik der Koronararterien, Lungenfunktion) sind in Abhängigkeit des Umfangs der geplanten Operation bzw. des Gesundheitsstatus der Patient:innen zu erheben [4, 15].

Im Rahmen der präoperativen Evaluierung und der Operationsvorbereitung ist aus diabetologischer Sicht eine funktionierende Informationsübermittlung und Kooperation zwischen vorbehandelnden Ärzt:innen, Chirurg:innen und Anästhesist:innen zu gewährleisten, da das gewählte Anästhesieverfahren einen wesentlichen Einfluss auf die erforderlichen präoperativen Befunde respektive die prä- bzw. perioperative Therapie hat [4, 6, 16]. Bei komplexen diabetologischen Therapieregimen und/oder beim Vorliegen diabetischer Spätsyndrome ist die Beiziehung von diabetologisch versierten Ärzt:innen geboten [17].

## Präoperative Stoffwechselkontrolle

Im Rahmen eines operativen Eingriffes kann es aufgrund der Auslenkung von antiinsulinär wirkenden endogenen Hormonen (Glukagon, Kortisol, Somatropin, Katecholamine) und des Auftretens von Entzündungsmediatoren im Rahmen der Akute-Phase-Reaktion zu einer Verschlechterung/Entgleisung einer diabetischen Stoffwechsellage kommen. Weiters kann eine Diabeteserkrankung unter diesen Umständen klinisch erstmanifestieren oder im Rahmen des präoperativen Evaluierens zufällig erstdiagnostiziert werden, weshalb die präoperative Evaluierung der glykämischen Stoffwechsellage relevant ist [6, 7, 9, 18–20]. Perioperative Hyperglykämie (> 140 mg/dl) ist bei Menschen mit und ohne Diabetes mellitus ein Risikofaktor für schlechtere postoperative Resultate und ist mit 20 bis 40 % bei allgemeinchirurgischen und 80 bis 90 % bei kardiochirurgischen Patient:innen häufig [21–25].

Menschen mit Diabetes mellitus weisen ein prinzipiell erhöhtes Risiko für Infektionen bzw. für postoperative Infektionskomplikationen auf [21, 26–31]. Eine Metaanalyse von 14 prospektiven Kohortenstudien (*N* = 91.094 Patient:innen) ergab eine signifikante Erhöhung des Risikos für postoperative Wundinfektionen bei Patient:innen mit Diabetes mellitus um 69 % (RR 1,69; 95 % Confidence Interval (CI), 1,33–2,13) [32]. Eine weitere Metaanalyse von 94 Studien, publiziert zwischen 1986 und 2015, ergab ebenfalls eine Erhöhung des Risikos für postoperative Wundinfektionen um 53 % (OR 1,53; 85 % Predictive Interval (PI) 1,11–2,12), wobei die Operationslokalisation, das Studiendesign und der Body-Mass-Index (BMI) der Patient:innen keinen Einfluss hatte [33]. Das Auftreten postoperativer Infektionen war aber mit Hyperglykämie vor (OR 1,88; PI 0,66–5,34) und nach der Operation (OR 1,45; 95 % 0,77–3,04) assoziiert. Die Art des operativen Eingriffs ist nicht nur für die präoperative Evaluierung, sondern auch für das postoperative Komplikationsrisiko entscheidend: Bei herzchirurgischen Eingriffen war das postoperative Infektionsrisiko bei Diabetes mellitus mit einer OR von 2,03 signifikant höher als bei den übrigen chirurgischen Eingriffen [34]. Auch das Auftreten postoperativer kardiovaskulärer Komplikationen ist bei Patient:innen nach kardialen Eingriffen signifikant erhöht [34]. Zusätzliche Risikofaktoren für Infektionen stellen ausgeprägte Adipositas bzw. mikro- und makroangiopathische Durchblutungsstörungen und das Vorliegen einer diabetischen Nephropathie dar [6, 35–37].

Erhöhte präoperative Blutzuckerwerte sind mit erhöhtem Risiko für postoperative Komplikationen assoziiert [38–40]. Daher sollten akute Blutzuckerentgleisungen mit Hyperglykämien über 300 mg/dl eine elektive Operation postponieren bis sich die Blutglukose zumindest für ein bis vier Stunden präoperativ im Zielbereich (zumindest < 180 mg/dl) befindet und Patient:innen metabolisch kompensiert sind [20, 39, 41].

Der HbA1c-Wert ist international als Surrogat Parameter der langfristigen glykämischen Kontrolle akzeptiert. Im perioperativen Kontext konnte eine Korrelation zu schlechteren postoperativen Ergebnissen bei erhöhten HbA1c-Werten gezeigt werden [42–47]. Es wird jedoch kontroversiell diskutiert, ob das HbA1c als zuverlässiger Prädiktor postoperativer Komplikationen tatsächlich geeignet oder ob nicht die akute perioperative Stoffwechsellage ein besserer Marker ist [48–52]. Eine retrospektive Subanalyse der BARI-2D-Studie ergab bei aortokoronaren Bypass-Operationen ein signifikant höheres Risiko für kardiovaskuläre Komplikationen (MACE) (HR 1,77) und instabile Angina Pectoris (HR 5,21) bei präoperativen HbA1c-Werten über 8,0 % (versus 6,1 bis 7,0 %), wohingegen ein HbA1c unter 6,0 % mit einem erhöhten Mortalitätsrisiko (HR 2,41) assoziiert war [53]. Bei bariatrischen Operationen gestaltet sich das Erreichen präoperativer HbA1c-Ziele schwierig und langwierig, weshalb in diesem Kollektiv Zielwerte differenziert betrachtet werden sollten [50, 52]. Dennoch ist prinzipiell eine klare Assoziation von chirurgischen Komplikationen zur vorbestehenden chronischen Stoffwechselkontrolle anzunehmen [30, 46, 54].

Präoperativ sollte aus Sicht der Autor:innen ein HbA1c-Wert von 7,0 % bzw. darunter angestrebt werden. Bei Patient:innen, bei welchen eine derartig strikte Stoffwechselkontrolle nicht erzielbar ist bzw. aufgrund von begleitender Multimorbidität und fortgeschrittenem Alter nicht geboten ist, sollte der HbA1c-Wert vor geplanten Operationen zumindest unter 8,0 % liegen. Operationen bei HbA1c-Werten von über 10,0 % sollten nur bei vitaler bzw. dringlicher Operationsindikation durchgeführt werden (Expert:innenmeinung, Evidenzlage C). Bei Patient:innen, bei denen ein HbA1c-Wert präoperativ unter 8,0 % nicht möglich erscheint, sollte die Blutglukose zumindest vier Stunden vor der Operation unter 180 mg/dl liegen, um postoperative Komplikationen zu minimieren [41]. Inwieweit moderne Zielparameter (z. B. Zeit im Zielbereich (TIR), Glukose Management Indikator (GMI) und Glukosevariabilität (GV)) zukünftig in die präoperative Einschätzung der Stoffwechsellage Einzug finden, ist in zukünftigen Studien zu evaluieren.

## Perioperative Stoffwechselkontrolle & Komplikationen

Im Krankenhaus können für ein strukturiertes perioperatives Management von Menschen mit Diabetes verschriftliche Standards qualitätsverbessernd sein [6, 55, 56]. Sinnvollerweise soll die Nüchternphase so kurz wie möglich gehalten werden und wenn möglich die Operation als erster Punkt im Operationsplan stattfinden [6, 28, 57]. In der Pro-Diab Melbourne Studie war ein strukturierter perioperativer Diabetes Management Plan vor Aufnahme bei elektiven Eingriffen mit einem sicheren Umgang antihyperglykämer Medikation sowie verbesserter perioperativer Glykämie assoziiert [58].

Die perioperative Stoffwechselkontrolle soll primär mittels kapillärer Blutzucker- oder venöser Plasmaglukosemessung monitorisiert werden. Die perioperative Messfrequenz ist abhängig von der Fähigkeit der Patient:innen im Selbstmanagement und der Vigilanz. Präoperativ soll während Nüchternheitsphasen zumindest alle zwei bis vier Stunden gemessen werden. Im Falle von intravenöser (i.v.) Insulinzufuhr empfiehlt sich eine Messung alle 30 bis 120 min [7].

Der Einsatz von CGM im intramuralen Bereich wird im Kapitel Diabetesmanagement im Krankenhaus diskutiert [59]. Für das perioperative Management sollten potenziell interferierende Faktoren der CGM-Messgenauigkeit (Hypothermie, Diathermie, Hypoxie, Medikamente, Durchblutung, Lagerung, chronische Nierenerkrankung) sowie die fehlende Zulassung in Österreich kommerziell erhältlicher Systeme für diese Indikation beachtet werden [7, 60–64]. In einem Kollektiv nach Kardiochirurgie (*N* = 60, 26,7 % mit Diabetes) konnte eine akzeptable CGM-Genauigkeit bei kurzer Nachbeobachtung von 23 h gezeigt werden [65]. Verglichen mit einem intravaskulärem Mikrodialyse CGM zeigte ein subkutanes CGM im Vergleich zur Referenzmethode bei kardiochirurgischen Patient:innen (*N* = 24, 25 % mit Diabetes) perioperativ wiederholt niedrigere Werte (mittlere absolute relative Differenz (MARD) 6,5 % versus 30,5 %) [66]. Ein Benefit der kontinuierlichen Messung (insbesondere von Systemen mit Alarmfunktion) perioperativ könnte in der Erkennung von in punktuellen Messungen unbemerkten Hypoglykämien liegen, die bereits außerhalb des perioperativen Kontexts mit erhöhter Mortalität einhergehen [12, 67]. Im Umkehrschluss birgt diese Situation jedoch auch eine rechtliche Problematik: Aufgrund fehlender telemetrischer Überwachungsmöglichkeit könnten Hypoglykämien zwar aufgezeichnet, aber vom ärztlichen Personal nicht wahrgenommen werden.

Ziele der perioperativen Glukosekontrolle sind das strikte Vermeiden schwerer Hypoglykämien und ausgeprägter hyperglykämischer Stoffwechselentgleisungen, da diese mit erhöhter Komplikationsrate sowie längerer Krankenhausaufenthaltsdauer und gesteigerter Mortalität assoziiert sind [6, 7, 21, 58, 68–71]. Eine stabile perioperative Einstellung des Blutzuckers ist relevant, um das perioperative Risiko zu minimieren [72, 73].

An dieser Stelle sei erwähnt, dass der Großteil des Kollektivs vorhandener Studien Menschen mit Diabetes mellitus Typ 2 (DMT2) einschloss, sodass diese Empfehlungen mangels Evidenz auch auf Menschen mit Diabetes mellitus Typ 1 (DMT1) übertragen werden. Eine striktere Einstellung unter Vermeidung von Hypoglykämien wäre – insbesondere bei DMT1 – möglicherweise sinnvoll [7, 12, 74]. Perioperativ sollen Blutzuckerwerte zwischen 110 und 140 mg/dl angestrebt werden, wobei ein Zielbereich von 80 bis 180 mg/dl als adäquat anzusehen ist [6, 7, 28, 75, 76]. Blutzuckerwerte über 180 mg/dl auf Intensivstationen bzw. über 200 mg/dl auf der Normalstation sind zu vermeiden und legen die Einleitung einer Insulintherapie nahe [7, 77]. Bezüglich der Güte der Stoffwechselkontrolle des (perioperativ) intensivmedizinisch zu betreuenden Patient:innen wird auf das ÖDG-Positionspapier „Therapie der Hyperglykämie bei kritisch kranken Patienten“ verwiesen [78].

Im Rahmen einer Operation kann es aufgrund einer gesteigerten katabolen Stoffwechsellage durch präoperative Nüchternheit zum Postaggressionssyndrom kommen. Durch die Ausschüttung von endogenen Stresshormonen ist mit einem erhöhten Risiko für postoperative Hyperglykämien bis potenziell lebensbedrohlichen diabetischen Ketoazidosen zu rechnen. Da Menschen mit Diabetes mellitus keine adäquate, körpereigene Gegenregulation besitzen, ist es insbesondere in der postoperativen Phase wichtig die Blutzuckerwerte engmaschig zu überwachen [6, 79].

Der Nutzen einer postoperativen nahe-normoglykämischen Blutzuckerkontrolle kritisch Kranker ist auf Basis von Metaanalysen prospektiver Studien nicht für alle Patient:innengruppen erwiesen [77]. Laut einer rezenten Übersichtsarbeit ist das erhöhte Risiko für postoperatives Delirium oder kognitive Dysfunktion mit chronischer und/oder perioperativer Hyperglykämie positiv assoziiert [80]. Eine Analyse klinischer Ergebnisse und medizin-ökonomischer Folgen ergab bei Patient:innen ohne Diabetes eine höhere Komplikationsrate, wenn postoperativ wiederholt Blutglukosewerte über 180 mg/dl gemessen wurden, während bei Menschen mit Diabetes mit vorbestehender Insulintherapie die geringsten postoperativen Komplikationen in hyperglykämischen Bereichen zwischen 180 und 240 mg/dl auftraten [81]. Analoge Ergebnisse zum Einfluss der perioperativen Glukosekontrolle ergab eine Cochrane Analyse aus dem Jahr 2012, welche bei Patient:innen unter intensivierter perioperativer glykämischer Kontrolle keine signifikante Verbesserung der chirurgischen Ergebnisse, aber eine erhöhte Hypoglykämierate fand [82]. In der randomisierten Leuven-Studie wurde bei beatmeten Patient:innen nach kardiochirurgischen Eingriffen – auch ohne Diabetes mellitus – eine strikte Blutglukoseeinstellung (80 bis 110 mg/dl) als günstig hinsichtlich der Mortalität eingestuft [83]. Die multizentrische NICE-SUGAR Studie dagegen ergab bei kritisch kranken Patient:innen keinen signifikanten Vorteil durch striktere glykämische Einstellung (81 bis 108 mg/dl versus 140 bis 180 mg/dl), sondern eine signifikant erhöhte Mortalität (27,5 % versus 25 %) [84]. Eine rezente Metaanalyse zeigte reduzierte postoperative Wundinfektionen bei strikteren Glukosezielbereichen für kardiochirurgische und allgemeinchirurgische Populationen, jedoch war dies einhergehend mit zunehmenden Hypoglykämien und erhöhter Mortalität [85]. Eine zu strikte normnahe perioperative Glukoseeinstellung ist möglicherweise wegen iatrogen bedingter Hypoglykämien nachteilig, da diese intramural wie perioperativ mit erhöhter Mortalität assoziiert sind [86, 87]. Für isolierte herzchirurgische Eingriffe hingegen scheinen günstige Ergebnisse (inklusive einer verringerten postoperativen Frühmortalität) für eine strikte perioperative Glukosekontrolle nachweisbar, solange diese ohne signifikant erhöhte Hypoglykämierate erzielt werden kann [25, 88, 89]. Dies wird auch durch eine rezente Analyse von über 7000 Patient:innen untermauert, bei welchen das Vorliegen eines Diabetes mellitus und höherer HbA1c-Werte als unabhängige Risikofaktoren mit einem schlechteren postoperativen Ergebnis, u. a. höherer 6 -Monate Mortalität, schwerwiegenden Komplikationen und einem längeren Spitalsaufenthalt assoziiert war [90]. Dies mag unter anderem dadurch bedingt sein, dass laut einer US-amerikanischen Studie mit mehr als 10 Mio. Patient:innen, welche sich einer nicht kardiochirurgischen Operation unterzogen hatten, perioperative kardiovaskuläre und zerebrovaskuläre Ereignisse bzw. Komplikationen bei Menschen mit Diabetes mellitus durchschnittlich um ca. 20 % höher lagen (3,3 versus 2,8 %, *p* < 0,001) und über den Beobachtungszeitraum im Gegensatz zu den nichtdiabetischen Patient:innen hinsichtlich der Frequenz zunahmen [91]. Zudem weisen Daten von über 80.000 Menschen mit Diabetes mellitus aus hausärztlichen Praxen in England darauf hin, dass sowohl DMT1 als auch DMT2 in Abhängigkeit von der Güte der glykämischen Kontrolle bei hohen HbA1c-Werten mit einem gesteigerten Risiko für das Auftreten von schwerwiegenden Infektionen und konsekutiver Mortalität assoziiert sind [92].

Septische Patient:innen auf Intensivstationen ohne vorbestehenden insulinbehandelten Diabetes mellitus zeigten bei ausgeprägter Hyperglykämie in den ersten 24 h nach Aufnahme eine erhöhte Mortalität, während diese bei insulinvorbehandelten Patient:innen – relativ gesehen – erniedrigt war [93]. Erhöhte Glukosekonzentrationen im Sinne einer „Stresshyperglykämie“ bei Menschen ohne manifeste Diabetesdiagnose sind möglicherweise mit einer erhöhten Rate an postoperativen Komplikationen und Spitalsmortalität verbunden [30]. Ob in diesem Kollektiv eine therapeutische Intervention und Glukosesenkung durch i.v. Insulingabe die Prognose verbessern kann, ist noch unklar und sollte Gegenstand zukünftiger Studien sein. Möglicherweise ist die stress-getriggerte Hyperglykämie hierbei Korrelat eines schweren Verlaufes [94]. Eine japanische Arbeitsgruppe zeigte rezent, dass zwischen dem postoperativen Insulinbedarf nach Spinalkanalstenose-Operation bei Menschen mit DMT2 eine positive Korrelation mit dem Akut-Phase-Protein C-reaktives Protein (CRP) bestehen könnte. In der Modellberechnung stieg der Insulinbedarf um 0,60 Internationale Einheiten (IE) täglich pro CRP-Anstieg um 1 mg/dL [95]. Eine zunehmende Insulinresistenz durch Inflammation und postoperative Stresshormone machen dies aus pathophysiologischer Sicht erklärbar [94, 96].

## Perioperative medikamentöse Diabetestherapie

Generell sollen orale Antidiabetika am Tag der Operation (zumeist morgens) pausiert werden. Für einzelne Wirkstoffklassen wird ein längeres Pausieren perioperativ empfohlen, was im Folgenden erläutert wird. Bei kurzen operativen Eingriffen kann die orale Therapie nach unkompliziertem chirurgischem Verlauf und Aufnahme der Nahrungszufuhr wieder angesetzt werden. Bei längeren Operationen sollte frühestens am ersten postoperativen Tag die orale antidiabetische Therapie zeitgleich mit der ersten oralen Nahrungszufuhr bzw. Beginn des oralen Nahrungsaufbaus wieder begonnen werden. Die folgenden Empfehlungen beziehen sich auf Menschen mit Diabetes mellitus und die entsprechende Indikation der genannten Substanzklassen, nicht aber auf weitere Anwendungsbereiche außerhalb der antidiabetischen Wirkung (insbesondere GLP1-RA und SGLT-2-Inhibitoren). Zum Diabetesmanagement im Krankenhaus abseits von Operationen wird auf das entsprechende Leitlinienkapitel verwiesen [59].

### Biguanide: Metformin

Für Metformin wird ein Absetzen am Tag der Operation mit Anästhesie (Allgemein‑, Spinal- oder Epiduralnarkose) laut Zulassungstext empfohlen, wobei eine allfällige Kumulation von Metformin aufgrund von Nierenfunktionseinschränkung bzw. Nierenversagen zu verhindern ist. Bei Nierenfunktionseinschränkung mit Akkumulationsgefahr soll die Einnahme präoperativ 24 bis 48 h pausiert werden. Vor Wiederbeginn muss eine stabile Nierenfunktion (glomeruläre Filtrationsrate (GFR) > 30 ml/min) laborchemisch bestätigt werden und entweder 48 h postoperativ vergangen oder eine orale Nahrungsaufnahme möglich sein [97]. Metformin verzögert den Abbau von Laktat in der Leber, welches sich bei größeren Operationen bzw. bei gastrointestinalen Eingriffen vermehrt bilden kann. Das Risiko für eine Laktatazidose ist insbesondere für andere Vertreter der Biguanide erhöht, während bei Metformin eine Inzidenz in einem Kollektiv ohne Akuterkrankung bei 0,03 bis 0,06 pro 1000 Patient:innenjahren beschrieben ist [98–100]. Bei normaler Nierenfunktion beträgt die Plasmahalbwertszeit von Metformin etwa 13 h. Bei sonst gesunden Menschen mit Diabetes genügt vor kleineren Eingriffen ein Pausieren von Metformin am Operationstag [7, 9, 16]. Jedenfalls sollte Metformin postoperativ bis zur Sicherstellung einer adäquaten Nierenfunktion pausiert bleiben. In einigen Publikationen wird das Fortführen von Metformin perioperativ empfohlen [28, 69, 101]. In einer randomisiert kontrollierten Studie konnte für nicht kardiochirurgische Eingriffe gezeigt werden, dass das perioperative Einnehmen von Metformin nicht mit verbesserter postoperativer Glukose oder signifikanten Unterschieden im Serumlaktat assoziiert ist [102].

### Inkretin-basierte Therapien

Dipeptidyl-Peptidase‑4 (DPP4) -Hemmer (Gliptine) und vor allem subkutan zu verabreichende Glucagon like Peptid 1‑Rezeptoragonisten (GLP1-RA) können die Magenentleerung verzögern und gastrointestinale Nebenwirkungen wie Übelkeit und Erbrechen auslösen [103]. In einem systematischen Review von randomisiert kontrollierten Studien zu Inkretin-basierten Therapien im perioperativen oder intensivmedizinischen Bereich ergaben sich Hinweise für niedrigere Blutglukosewerte ohne signifikante Zunahme von Hypoglykämien [104]. Der potenziell vorteilhafte Einsatz von DPP4-Hemmern und GLP-1-RA bei Herz-Thoraxchirurgie postoperativ wird postuliert, wobei weitere großangelegte Studien gefordert werden [105].

#### DPP4-Hemmer

In einer prospektiven, randomisiert-multizentrischen Studie wurde bei Menschen mit DMT2 mit nicht kardiochirurgischen Eingriffen die Wirksamkeit und Sicherheit von Linagliptin im Vergleich zu Basis-Bolus-Therapie verglichen. In diesem Kollektiv zeigte sich eine reduzierte Rate an Hypoglykämien (86 % relative Risikoreduktion), wobei in einer Subgruppenanalyse von Patient:innen mit Blutzuckerwerten über 200 mg/dl die mittlere Glukose mit Linagliptin höher war als in der Vergleichsgruppe [106]. Eine andere randomisierte Studie zeigte bei Patient:innen ohne Diabetes mit allgemeinchirurgischen Operationen, dass postoperative Stresshyperglykämie nicht durch Therapie mit Sitagliptin vermieden werden konnte [107]. Ähnliche Ergebnisse zeigte eine verblindete placebo-kontrollierte Studie mit Sitagliptin bei Menschen mit DMT2 und kardiochirurgischen Eingriffen: Postoperative Hyperglykämie konnte in diesem Kollektiv nicht reduziert werden [108]. In der prospektiven randomisierten Sita-Hospital Untersuchung konnte für Sitapliptin plus Basalinsulin keine Unterlegenheit hinsichtlich der glykämischen Kontrolle im Vergleich zu Basis-Bolus-Therapie bei DMT2 gezeigt werden [109]. Aufgrund der Abwesenheit von perioperativer Hypoglykämiegefahr ist die perioperative Fortführung von DPP4-Hemmern möglicherweise indiziert, aufgrund mangelnder Evidenz ist ein Pausieren am Tag der Operation empfehlenswert [7, 110].

#### GLP1-Rezeptoragonisten

In der placebo-kontrollierten GLOBE Studie an kardiochirurgischen Patient:innen mit und ohne DMT2 zeigte eine präoperative Gabe von Liraglutid einen reduzierten i.v. Insulinbedarf und verbesserte glykämische Kontrolle [111]. Auch die GLOLIA Studie, in der Liraglutid bei Kardiochirurgie als Kombinationstherapie zu Insulin bei Menschen mit DMT2 eingesetzt wurde, ergab perioperativ Hinweise für eine verbesserte Glykämie [112]. In einem Kollektiv von Menschen mit DMT2 und nicht kardiochirurgischen Eingriffen konnte eine randomisiert-kontrollierte Studie mit Liraglutid (versus Glukose-Insulin-Kalium-Infusion oder i.v. Insulinbolus) perioperativ stabilere Glukosewerte und reduzierten Bedarf an zusätzlichem Insulin zeigen, wobei die Rate an präoperativer Übelkeit zunahm [113]. Das PILGRIM-Studienprotokoll soll den perioperativen Einsatz von GLP1-RA bei Patient:innen mit DMT2 untersuchen, wobei bis 2022 keine Ergebnisse publiziert wurden [114]. Der perioperative Einsatz von GLP1-RA ist somit nicht ausreichend durch die vorhandene Datenlage gestützt, eine Fortführung ist aus Sicht der Autor:innen denkbar, sofern keine positive Anamnese für postoperative Übelkeit und Erbrechen (PONV) vorliegt [57]. Die Entscheidung diesbezüglich sollte interdisziplinär mit den behandelnden Anästhesist:innen getroffen werden.

### Sodium Glukose Co-Transporter-2 (SGLT-2)-Inhibitoren

Aufgrund pharmakodynamischer Effekte der SGLT-2-Inhibitoren (Gliflozine), kommt es zu einer gesteigerten Ketonreabsorption und iatrogen-induzierter Glukosurie [115, 116]. In Kombination mit relativem oder absolutem Insulinmangel mit daraus resultierender gesteigerter Ketogenese, kann es zur seltenen Nebenwirkung der euglykämen diabetischen Ketoazidose (EDKA) kommen, die aufgrund des Mangels pathognomonischer Symptome und potenzieller Abwesenheit von relevanter Hyperglykämie verzögert erkannt werden kann. In der Literatur wurden Risikofaktoren für EDKA identifiziert: Stress, Infektion, akute Erkrankung, Insulindosisreduktion, Nüchternheit und perioperativer Kontext wurden hierbei als potenzielle Trigger beschrieben [116–120]. Hinsichtlich der Art der Operation scheinen bariatrische und kardiochirurgische Eingriffe als besonders risikobehaftet hinsichtlich EDKA [121–125]. Während in einer rezenten Metaanalyse der Zulassungsstudien der Gliflozine mit bewiesenem kardiorenalen Benefit unter randomisiert-kontrollierten Bedingungen kein erhöhtes Risiko für EDKA beschrieben wurde, zeigt sich in real-world Populationen eine tendenziell höhere Frequenz im perioperativen Kontext [126, 127]. Die SAPKA-Studie wird prospektiv an Menschen mit DMT2 und Eingriffen in Vollnarkose die Inzidenz von postoperativer EDKA bei Fortführen von SGLT2-Inhibitoren evaluieren [128]. Ein Review aus anästhesiologischer Sicht, der 42 Fälle von EDKA unter Gliflozintherapie zusammenfasst, beschreibt 12 Fälle nach bariatrischen Operationen unter hypokalorischer Kost, aber keinen klaren zeitlichen Zusammenhang zwischen dem Zeitpunkt des Absetzens des SGLT-2-Hemmers und des Auftretens der Ketoazidose. Hierbei wird vor einer verzögerten Diagnosestellung aufgrund der untypischen Präsentation gewarnt [121]. Da SGLT-2-Hemmer immer breitere Verwendung finden und auch bei chirurgischen Patient:innen ohne Diabetes mit Herzinsuffizienz und/oder chronischer Nierenerkrankung Anwendung finden, erscheint eine adäquate Medikamentenanamnese besonders wichtig.

SGLT-2-Inhibitoren haben eine Halbwertszeit von 8 bis 16 h, weshalb ein präoperatives Pausieren von 3 bis 5 Tagen rational begründbar ist [123, 129–131]. Internationale Empfehlungen einschlägiger Fachgesellschaften sind aufgrund der mangelnden Datenlage uneinheitlich [7, 132–134]. Aus Sicht der Autor:innen sollten SGLT-2-Inhibitoren bei Menschen mit DMT2 und langdauernden Operationen (> 2 h), mit erwartbarer postoperativer Nahrungskarenz, bei größeren Eingriffen in Allgemeinnarkose – insbesondere bei Kardiochirurgie – sowie bei perioperativer Insulindosisreduktion optimalerweise 72 h präoperativ pausiert werden. Ein Wiederansetzen darf postoperativ erst bei stabiler kardiovaskulärer und metabolischer Situation erfolgen. Auch bei kurzdauernden Eingriffen in Regionalanästhesie ist aus Sicherheitsgründen ein Pausieren der SGLT2-Inhibitoren von zumindest 48 h präoperativ empfehlenswert [7, 135–137]. Bei notfallmäßigen Operationen hingegen soll die SGLT2-Inhibitor Einnahme so zeitnahe als möglich pausiert und perioperativ die metabolische Kontrolle und Möglichkeit der seltenen Nebenwirkung einer EDKA beachtet werden [110]. Wenn SGLT-2-Inhibitoren präoperativ 72 h pausiert werden, ist eine Hyperglykämie (> 180 mg/dl) vor der Operation kurzfristig denkbar. Internationale Leitlinien und Fachgesellschaften geben diesbezüglich noch keine klaren Handlungsempfehlungen ab, es wird jedoch empfohlen andere antidiabetische Medikation gegebenenfalls zu erhöhen [133]. Aus dem Mangel verfügbarer Evidenz diesbezüglich, wird bei perioperativer Hyperglykämie (> 180 mg/dl) der Einsatz von kurzwirksamen Insulinanaloga subkutan zur Korrektur empfohlen. Diese Empfehlung wird auch daraus abgeleitet, dass derzeit kein auf dem Markt verfügbares orales antidiabetisches Medikament zur perioperativen antihyperglykämischen Therapie tatsächlich empfohlen ist. Jedenfalls sollte das perioperative Management der Gliflozine nach individuellem Ermessen in Abhängigkeit der Indikation (Diabetes mellitus und/oder Herzinsuffizienz und/oder chronische Nierenerkrankung), Diabeteseinstellung und Therapieregime (Insulintherapie versus orale antidiabetische Therapie) sowie Operationsart und -schwere interdisziplinär mit dem Team der Anästhesie festgelegt werden.

### Peroxisom-Proliferator-aktivierter Rezeptor (PPAR)-y Agonist: Pioglitazon

Pioglitazon kann vermehrte Flüssigkeitsretention begünstigen und somit zur Volumenüberlastung beitragen. Aufgrund von mangelnder Evidenz wird entsprechend der Expert:innenmeinung das Pausieren von Glitazonen am Operationstag empfohlen [138].

### Insulinsekretagoga: Sulfonylharnstoffe und Glinide

Sulfonylharnstoffe und Glinide können bei mangelnder Nahrungszufuhr (z. B. 12-stündiges Fasten präoperativ) Hypoglykämien auslösen. Zudem deuten tierexperimentelle Studien auf eine mögliche ungünstige Interferenz auf Hypoxie-bedingte Vasodilatation hin, was z. B. bei Patient:innen mit kritischer Koronardurchblutung Probleme verursachen könnte [139]. Es herrscht in der vorhandenen Literatur einheitlich die Empfehlung, Sekretagoga am Tag einer Operation zu pausieren bis eine orale Nahrungszufuhr wieder gewährleistet ist [57, 110, 138, 140].

### Insulintherapie

Die klare Kennzeichnung und Dokumentation des Diabetestyps (insbesondere DMT1, DMT2, Diabetes mellitus Typ 3 (DMT3) und Gestationsdiabetes) im perioperativen Kontext ist sinnvoll und wichtig, da insbesondere übergewichtige/adipöse Menschen mit DMT1 und DMT3 fälschlicherweise als Menschen mit DMT2 behandelt werden könnten [6, 74]. Es soll an dieser Stelle Erwähnung finden, dass perioperatives Auslassen einzelner Insulindosen, speziell bei Menschen mit DMT1, gravierende Akutkomplikationen nach sich ziehen kann. Die Angst vor prä- oder postoperativen Hypoglykämien und eine daraus resultierende ausgelassene Insulingabe kann bei DMT1 zu potenziell lebensbedrohlichen perioperativen diabetischen Ketoazidosen führen. Daher muss bei diesem Kollektiv das vorrangige perioperative Ziel sein, eine durchgängig adäquate Basalinsulinzufuhr zu gewährleisten, die eine stabile Stoffwechsellage ermöglicht und diabetische Akutkomplikationen vermeidet. Ein präoperativer Nüchternblutzucker im normoglykämen Zielbereich darf kein Grund für eine unzureichende Substitution des Basalinsulins mit konsekutiven absoluten, perioperativen Insulinmangelzuständen sein. Sollte es Hinweise für eine präoperativ zu hohe Basalinsulindosis geben (anamnestisch gehäufte Hypoglykämien), kann eine Dosisreduktion am Vorabend oder Tag der Operation sinnvoll sein. Im Optimalfall ist präoperativ jedoch bereits eine Evaluierung und gegebenenfalls Anpassung der Basaldosis (z. B. Basalratentest, CGM-Verlauf über Nacht) erfolgt [7, 59, 74, 138, 141].

Insulinpräparate sind vor allem bei schweren und längeren Eingriffen mit protrahierter intensivmedizinischer Betreuung derzeit in der Regel die einzige therapeutische Option, perioperativ die Blutzuckerwerte zu kontrollieren [9, 16, 18]. Die randomisierte Multizenterstudie Rabbit 2 Surgery verglich Basis-Bolus-Therapie (Insulin Glargin und Glulisin) mit Korrektur durch Humaninsulin nach Schema vier Mal täglich hinsichtlich der Güte der Blutzuckereinstellung und postoperativer Komplikationen (Composite aus Wundinfektion, Pneumonie, Bakteriämie, Nieren- und respiratorisches Versagen) bei allgemeinchirurgischen Patient:innen mit DMT2. Hierbei zeigte sich, dass die mittlere Blutglukose gesenkt und der zusammengesetzte Endpunkt mit Basis-Bolus-Therapie signifikant seltener eintrat [142]. Auch eine später durchgeführte randomisierte Studie bestätigte die Überlegenheit von Basal-Bolus-Therapie oder Basal-Plus-Therapie (Basalinsulin und zusätzliche Dosen von kurzwirksamem Insulin Glulisin versus Korrektur durch Humaninsulin nach Schema) bei nicht-kardiochirurgischen Patient:innen mit DMT2 [143].

Bei großen Operationen mit protrahierter intensivmedizinischer Betreuung ist eine an aktuell gemessene Blutglukosewerte adaptierte i.v. Verabreichung von kurzwirksamen Insulinanaloga die Therapie der Wahl. In der Regel sind Insulindosen von 1 bis 3 IE Insulin pro Stunde ausreichend, um die Blutglukose zu kontrollieren. Empfehlenswert ist die gleichzeitige Bereitstellung von i.v. Glukoseinfusionen (ggf. mit Kaliumzusatz), um hypoglykämische Werte rasch korrigieren zu können [144, 145]. Ab Beginn einer (i.v.) Insulintherapie sollte der Zielbereich der Blutglukose 140 bis 180 mg/dl sein.

Bei Patient:innen mit basalunterstützter oraler Therapie (BOT) kann bei Routineoperationen das abendliche bzw. morgendliche Basalinsulin in unveränderter Dosis appliziert werden, nachdem die orale Therapie (s. oben) präoperativ pausiert wurde. Engmaschige Blutglukose-Kontrollen perioperativ sind erforderlich, um allfällige Korrekturen mittels i.v. Glukoseinfusion bzw. subkutaner zusätzlicher Gabe von raschwirksamem Insulin bzw. Insulinanaloga zu gewährleisten. Eine Dosisreduktion des Basalinsulins um 20–25 % kann bei präoperativ eher niedrigen Blutzuckerwerten sinnvoll sein [21, 69, 146].

Patient:innen mit Basis-Bolus-Insulintherapie sollen bei Routineoperationen die vorgesehene Basalinsulindosis applizieren. Korrekturen der Blutglukosewertewerte erfolgen in Abhängigkeit von engmaschig durchgeführten Kontrollen mittels Glukoseinfusion oder kurzwirksamen Insulins [6, 144, 145].

Als Faustregel ist davon auszugehen, dass das Kohlenhydratäquivalent einer Kohlenhydrateinheit (KE = 10 g Kohlenhydrate [147]) den Blutglukosewert um 25 bis 50 mg/dl hebt, eine zusätzlich gespritzte Einheit kurzwirksames Insulin den Blutzucker um 25 bis 50 mg/dl senkt (in Abhängigkeit von Insulinresistenz, Verteilungsvolumen und wirksamer Diabetestherapie). Somit müssen pro peroral oder intravenös zugeführter KE etwa 1 bis 2 IE kurzwirksamen Insulins (prandiales Insulin) zusätzlich zum Basalinsulin verabreicht werden, um eine Euglykämie zu gewährleisten.

Patient:innen unter konventioneller Insulintherapie mit einem Mischinsulin sollen bei Routineoperationen auf ein langwirksames Insulin (1 oder 2 × täglich gespritzt) umgestellt werden, wobei die zu veranschlagende Insulindosis des langwirksamen Insulins etwa zwei Drittel der Standarddosis des ursprünglichen Mischinsulins betragen soll. Entsprechende Korrekturen mit i.v. Glukose und kurzwirksamem Insulin sind wie oben dargestellt durchzuführen [21].

### Perioperative Insulinpumpentherapie & Künstliche Pankreassysteme

Bei großen bzw. langdauernden (> 2 h) Operationen sollten Patient:innen unter Insulinpumpentherapie (kontinuierliche subkutane Insulininfusion, CSII) perioperativ mittels einer i.v.-Insulininfusionstherapie behandelt werden. Bei kurzdauernden (< 2 h), elektiven Eingriffen ist eine Fortführung der CSII denkbar [21, 148]. Die Steuerung der von Patient:innen benutzten Insulinpumpe kann für nichtversierte Personen komplex sein [149]. Die vorbekannte Basalrate der Pumpe kann als Maßstab für die erforderliche Dosis an kurzwirksamen Insulinanaloga pro Stunde für die i.v.-Infusion herangezogen werden. Alternativ ist die Umstellung präoperativ von einer Insulinpumpe auf eine Basis-Bolus-Therapie anzudenken, wobei am Operationstag lediglich das Basalinsulin verabreicht wird [21, 148]. In Abhängigkeit von der Schwere und Dauer des geplanten Eingriffs, Vigilanz der Patient:innen und potenziellen Flüssigkeitsshifts ist eine Fortführung der CSII perioperativ denkbar. Eine klare Empfehlung zur Fortführung kann aus der derzeitigen Evidenzlage nicht abgeleitet werden, weshalb hier zu einer individuellen Entscheidung geraten wird [59, 148, 150]. Retrospektive Ereignisse einer chinesischen Arbeitsgruppe zeigten, dass eine CSII perioperativ die Glukosekontrolle versus Insulinspritzentherapie verbesserte und die postoperativen Infektionen verringerte, wobei der medizinische Gesamtaufwand nicht zunahm [151]. Ein aktuell publizierter Review postulierte die klinische Praktikabilität und Sicherheit einer fortgesetzten perioperativen subkutanen Insulininfusion unter bestimmten Rahmenbedingungen prinzipiell, wobei weitere prospektive Studien eingefordert werden [152]. Der perioperative Stellenwert von subkutanen automatisierten Insulinzufuhrsystemen (automated insulin delievery, AID) bzw. Hybrid closed-loop Systemen ist unzureichend durch Evidenz belegt. Hier soll jedenfalls erwähnt werden, dass der Einsatz von Hybrid closed-loop Systemen im intramuralen Bereich wie auch bei Dialysepflichtigkeit im Rahmen von Studien erfolgte und in diesen Kollektiven mit einer verbesserten glykämischen Kontrolle assoziiert war [153, 154].

Bezüglich künstlicher Intelligenz (AI) im perioperativen Kontext gibt es rezent interessante Arbeiten: In Japan wird an sogenannten Bedside-Pankreassystemen geforscht, die perioperativ mittels AI Insulin und Glukose intravenös zuführen und in dieser Indikation eine stabile glykämische Kontrolle und reduzierte postoperative Infektionsraten zeigen konnten [155]. Die postoperative glykämische Kontrolle bei Herz-Thoraxchirurgie konnte in einer weiteren Arbeit mittels Bedside-AI verbessert werden, wobei die Ergebnisse unabhängig vom Diabetesstatus des eingeschlossenen Kollektivs waren [156]. Es wird darüber hinaus postuliert, dass durch den perioperativen Einsatz des oben angeführten künstlichen Pankreassystems postoperative Komplikationen reduziert werden könnten [157, 158]. Eine andere Studie zeigte bei einem kardiochirurgischen Kollektiv, dass automatisierte intravenöse Insulinzufuhr die Patient:innen im glykämischen Zielbereich halten konnte ohne Hypoglykämien zu erhöhen [159].

## Zusatzmedikation

Aufgrund der häufig bestehenden Multimorbidität stehen viele Menschen mit Diabetes mellitus unter Begleittherapie mit Herzkreislauf-wirksamen Medikamenten (z. B. Präparate, welche das Renin-Angiotensin-System beeinflussen bzw. Betablocker) und unter Therapie mit gerinnungshemmenden Medikamenten (z. B. Thrombozytenaggregationshemmer in dualer Therapie, neue orale Antikoagulationen (NOAKs), Vitamin-K-Antagonisten, niedermolekulare Heparine usw.). Die Umstellung bzw. Pausierung dieser Medikamente ist in enger Absprache mit den behandelnden Teams der Chirurgie und Anästhesiologie zu bestimmen und muss sich am geschätzten Nutzen-Risiko-Profil für die Patient:innen orientieren. Im Zweifelsfall sind spezialisierte Ärzt:innen (z. B. Kardiolog:innen bei Zustand nach Stentimplantation) vor elektiven Eingriffen beizuziehen, um einerseits den günstigsten Operationstermin zu wählen bzw. ein optimales Management der gerinnungshemmenden Therapie festzulegen [4]. Einzelne Studien zu einer perioperativen Betablocker- bzw. Statintherapie werden kontroversiell diskutiert und lassen zum derzeitigen Stand keine klare Empfehlung zu [160–164].

Das international gängige Akronym „SADMANS“ umfasst Medikamentenklassen (Sulfonylharnstoffe, ACE-Inhibitoren, Diuretika, Metformin, Angiotensinrezeptor-Blocker, Nichtsteroidale Antirheumatika (NSAR), SGLT2-Inhibitoren), die im Krankheitsfall als sogenannte „Sick Day Drugs“ pausiert werden sollten. Aus Sicht der Autor:innen ist auch im perioperativen Setting eine Pausierung dieser Klassen sinnvoll und das Akronym eine praktische Merkhilfe für die Aufklärung von Patient:innen. Jedoch sollte das präoperative Pausieren von Medikamenten immer interdisziplinär entschieden und jedenfalls Inhalt der präoperativen Anästhesievisite sein [4, 165, 166].

## Empfehlungen

1. Menschen mit Diabetes mellitus haben ein höheres Risiko für Begleiterkrankungen als gleichaltrige Menschen ohne Diabetes. Das Risiko für Multimorbidität steigt allgemein mit dem Lebensalter und insbesondere mit der Diabetesdauer. Zu erwartende Begleiterkrankungen betreffen insbesondere das kardiovaskuläre System, Nieren, die Nerven und Sinnesorgane. Weiters bestehen häufig Zusatzerkrankungen im Sinne des metabolischen Syndroms (Empfehlungsgrad I, Evidenzklasse A).
2. Präoperative Untersuchungen sind in Abhängigkeit vom Umfang der geplanten Operation bzw. des Gesundheitsstatus der Patient:innen in enger Kooperation mit Anästhesist:innen und Chirurg:innen zu erheben (Empfehlungsgrad I, Evidenzklasse C).
3. Präoperativ sollte prinzipiell ein HbA1c-Wert von 7 % bzw. darunter angestrebt werden. Bei Patient:innen, bei welchen eine derartig strikte Stoffwechselkontrolle nicht erzielbar bzw. aufgrund von begleitender Multimorbidität und fortgeschrittenem Alter nicht geboten ist, sollte der HbA1c-Wert vor geplanten Operationen zumindest unter 8 % liegen. Operationen bei HbA1c-Werten von über 10 % sollten nur bei vitaler bzw. dringlicher Operationsindikation durchgeführt werden (Empfehlungsgrad IIb, Evidenzklasse C).
4. Generell sollen orale Antidiabetika am Tag der Operation (zumeist morgens) pausiert werden. Für Metformin wird bei Menschen mit Diabetes ohne Nierenfunktionseinschränkung ein Pausieren am Operationstag empfohlen, bei Nierenfunktionseinschränkung mit Akkumulationsgefahr 24 bis 48 h präoperativ. SGLT‑2 Inhibitoren bei Menschen mit DMT2 sollen vor größeren, langdauernden Eingriffen in Allgemeinnarkose sowie bei perioperativer Insulindosisreduktion 72 h präoperativ, bei kurzdauernden Eingriffen in Regionalanästhesie zumindest 48 h und bei notfallmäßigen Operationen so zeitnah wie möglich pausiert werden. Ein Wiederbeginn der Therapie ist erst nach Stabilisierung der klinischen Akutsituation, Abklingen allfälliger Entzündungen und Normalisierung der Nierenfunktion zulässig. Bei kurzen operativen Eingriffen kann die orale Therapie nach unkompliziertem chirurgischen Verlauf und Aufnahme der Nahrungszufuhr wieder angesetzt werden. Bei längeren Operationen sollte frühestens am ersten postoperativen Tag die orale antidiabetische Therapie wiederverordnet werden. Eine Kontrolle der Nierenfunktionsparameter vor neuerlicher Gabe von Metformin ist dabei erforderlich (Empfehlungsgrad IIa, Evidenzklasse C).
5. Insulinpräparate sind perioperativ (vor allem bei schweren und längeren Eingriffen mit protrahierter intensivmedizinischer Betreuung) derzeit die einzige therapeutische Option, um Blutzuckerwertewerte zu kontrollieren (Empfehlungsgrad I, Evidenzklasse C).
6. Ziele der perioperativen Glukosekontrolle sind das strikte Vermeiden schwerer Hypoglykämien und ausgeprägter hyperglykämischer Stoffwechselentgleisung mit Blutglukosewerten zwischen 80 und 180 mg/dl. Bei kritisch Kranken (auf Intensivstationen) erfordern Blutglukosewerte über 180 mg/dl die Initialisierung einer kontinuierlichen, intravenösen Insulintherapie, unter welcher in weiterer Folge die Blutglukose zwischen 140 und 180 mg/dl gehalten werden soll (Empfehlungsgrad I, Evidenzklasse A).
7. Auf Normalstationen sollen perioperativ Blutzuckerwerte zwischen 80 und 180 mg/dl angestrebt werden. Blutzuckerwerte über 180 mg/dl sind zu vermeiden bzw. über 200 mg/dl mittels Insulingabe zu therapieren (Empfehlungsgrad IIa, Evidenzklasse C).
8. Der Einsatz von kontinuierlichen Glukosemesssystemen (CGM) im perioperativen Management kann derzeit nicht generell empfohlen werden. Potenziell interferierende Faktoren der CGM-Messgenauigkeit sowie die fehlende Zulassung in Österreich kommerziell erhältlicher Systeme für diese Indikation müssen beachtet werden. Ein Vorteil könnte bei Systemen mit Alarmfunktion in der Erkennung von bei punktuellen Messungen unerkannten Hypoglykämien liegen. (Empfehlungsgrad IIb, Evidenzklasse C)
9. Bezüglich der perioperativen Insulinpumpentherapie (CSII) soll immer in Abhängigkeit von der Schwere und Dauer des geplanten Eingriffs, Vigilanz der Patient:innen und potenziellen Flüssigkeitsverschiebungen entschieden werden. Bei großen bzw. langdauernden (> 2 h) Operationen soll auf eine intravenöse Insulininfusion umgestellt werden, während bei kurzdauernden (< 2 h), elektiven Eingriffen eine Fortführung der CSII denkbar ist. (Empfehlungsgrad IIa, Evidenzklasse C)
10. Der perioperative Stellenwert von subkutanen automatisierten Insulinzufuhrsystemen (automated insulin delievery, AID) bzw. Hybrid closed-loop Systemen ist derzeit unzureichend durch Evidenz belegt. Auf individueller Entscheidungsbasis mit den behandelnden Teams der Anästhesiologie und Chirurgie werden im klinischen Alltag vorhandene Systeme bereits teilweise belassen und fortgeführt. (Empfehlungsgrad IIb, Evidenzklasse C)
11. Der Einsatz künstlicher Intelligenz (AI) könnte zukünftig im perioperativen Diabetesmanagement (z. B. Bedside-Pankreassysteme mit stabiler glykämischer Lage) denkbar sein, jedoch ist aus derzeitiger Evidenzlage und Zulassungssituation eine Empfehlung nicht möglich. (Empfehlungsgrad IIb, Evidenzklasse C)

## Abkürzungen

AGP: ambulantes Glukose Profil
AID: automatisierte Insulinzufuhrsysteme
AI: künstliche Intelligenz
BMI: Body Mass Index
BOT: basalunterstützte orale Therapie
CRP: C‑reaktives Protein
CGM: kontinuierliches Glukosemesssystem
CI: Confidence Interval
CSII: kontinuierliche subkutane Insulininfusion, Insulinpumpentherapie
DMT1: Diabetes mellitus Typ 1
DMT2: Diabetes mellitus Typ 2
DMT3: Diabetes mellitus Typ 3
DPP4: Dipeptidyl-Peptidase‑4
EDKA: euglykäme diabetische Ketoazidose
GFR: glomeruläre Filtrationsrate
GLP1-RA: Glucagon like Peptid 1‑Rezeptoragonisten
GV: Glukosevariabilität
GMI: Glukose Management Indikator
HbA1c: Hämoglobin A1c
HR: Hazard Ratio
IE: Internationale Einheit
i.v.: intravenös
MARD: mittlere absolute relative Differenz
NOAK: neue orale Antikoagulationen
NSAR: Nichtsteroidale Antirheumatika
OR: Odds Ratio
PI: Predictive Interval
PONV: Postoperative Übelkeit und Erbrechen
PPAR‑y: Peroxisom-Proliferator-aktivierter Rezeptor-y
SGLT‑2: Sodium Glukose Co-Transporter‑2
TIR: Zeit im Zielbereich
